# Herpes Simplex Virus 1-Induced Blood-Brain Barrier Damage Involves Apoptosis Associated With GM130-Mediated Golgi Stress

**DOI:** 10.3389/fnmol.2020.00002

**Published:** 2020-01-24

**Authors:** Qiang He, Hui Liu, Chuxin Huang, Renchun Wang, Minhua Luo, Wei Lu

**Affiliations:** ^1^Department of Neurology, The Second Xiangya Hospital, Central South University, Changsha, China; ^2^The Second Clinical Medicine School, Lanzhou University, Lanzhou, China; ^3^State Key Laboratory of Virology, CAS Center for Excellence in Brain Science and Intelligence Technology, Wuhan Institute of Virology, Chinese Academy of Sciences, Wuhan, China

**Keywords:** herpes simplex virus 1, herpes simplex encephalitis, blood-brain barrier, golgi apparatus, GM130, golgi stress, apoptosis

## Abstract

Herpes simplex encephalitis (HSE) caused by herpes simplex virus 1 (HSV-1) infection can lead to a high mortality rate and severe neurological sequelae. The destruction of the blood-brain barrier (BBB) is an important pathological mechanism for the development of HSE. However, the specific mechanism underlying the BBB destruction remains unclear. Our previous study found that the Golgi apparatus (GA) plays a crucial role in maintaining the integrity of the BBB. Therefore, this present study aimed to investigate the role of the GA in the destruction of the BBB and its underlying mechanisms. Mouse brain endothelial cells (Bend.3) were cultured to establish a BBB model *in vitro*, and then infected with HSV-1. The results showed that HSV-1 infection caused downregulation of the Golgi-associated protein GM130, accompanied by Golgi fragmentation, cell apoptosis, and downregulation of tight junction proteins occludin and claudin 5. Knockdown of GM130 with small interfering RNA in uninfected Bend.3 cells triggered Golgi fragmentation, apoptosis, and downregulation of occludin and claudin 5. However, overexpression of GM130 in HSV-1 infected Bend.3 cells by transient transfection partially attenuated the aforementioned damage caused by HSV-1 infection. When the pan-caspase inhibitor Z-VAD-fmk was used after HSV-1 infection to inhibit apoptosis, the protein levels of GM130, occludin and claudin 5 were partially restored. Taken together, these observations indicate that HSV-1 infection of Bend.3 cells triggers a GM130-mediated Golgi stress response that is involved in apoptosis, which in turn results in downregulation of occludin and claudin 5 protein levels. Meanwhile, GM130 downregulation is partially due to apoptosis triggered by HSV-1 infection. Our findings reveal an association between the GA and the BBB during HSV-1 infection and identify potentially novel targets for protecting the BBB and therapeutic approaches for patients with HSE.

## Introduction

Herpes simplex encephalitis (HSE) is the most common sporadic acute encephalitis worldwide that is often caused by herpes simplex virus type 1 (HSV-1), a ubiquitous, neurotropic, DNA enveloped virus (Whitley and Gnann, 2007; Granerod et al., 2010). Following the primary infection of mucosal or skin epithelium, HSV-1 will establish and maintain a life-long latency in the olfactory bulb, trigeminal ganglia, and other tissues. When the body’s immunity declines, the latent HSV-1 is reactivated and enters the central nervous system (CNS) *via* retrograde axonal transport, leading to the occurrence of HSE (Shivkumar et al., 2013; Tsalenchuck et al., 2014).

It is generally believed that HSV-1 causes brain damage in two ways. First, the virus activates the intracranial immunoinflammatory reaction, leading to immunopathological damage of brain tissue. Second, viral replication and its toxic products cause neuron and glia apoptosis (DeBiasi et al., 2002; Marques et al., 2006). In addition, Liu et al. (2019a,b) suggested that the destruction of the blood-brain barrier (BBB) is an important pathological mechanism for the development of HSE. After HSV-1 infection, chemokine receptors, leukocyte adhesion proteins, matrix metalloproteins, and inflammatory markers are significantly up-regulated, and the integrity of the BBB is affected (Pasieka et al., 2011). The permeability of the damaged BBB increases (Buursma et al., 2005), which results in the formation of brain edema, the influx of leukocytes, and the entry of various neurotoxic agents into the brain parenchyma to aggravate brain tissue injury (Keep et al., 2008; Moretti et al., 2015). However, the specific mechanism underlying the BBB damage during HSV-1 infection remains unclear.

The BBB is a physical border that separates the circulating blood from the brain and is formed by brain microvascular endothelial cells, endothelial cell-to-cell tight junction proteins, astrocytes, pericytes, and the basement membrane (Zhao et al., 2015). As a protective barrier for the CNS, the structural integrity and function of the BBB are crucial for the maintenance of CNS homeostasis (Abbott et al., 2010). In various neurological diseases, the BBB protects the CNS from toxins, pathogens, and inflammatory cytokines to a certain extent (Spindler and Hsu, 2012; Daneman and Prat, 2015). Tight junction proteins are important components of the BBB and they include claudin 5 and occludin. Many studies have found that changes in the expression levels and configuration of claudin 5 and occludin can directly affect the function of the BBB (Zlokovic, 2008; Chen et al., 2014; Chang et al., 2015). Nevertheless, there is still a lack of relevant studies that have investigated the effects of HSE on the expression and configuration of claudin 5 and occludin.

The Golgi apparatus (GA) is a crucial cytoplasmic organelle that acts not only as a key trafficking and sorting station and vital biosynthetic center for glycoproteins and lipids but also as an active signaling hub (Jiang et al., 2011; Zappa et al., 2018). Similar to the endoplasmic reticulum and mitochondria, the GA can initiate the signal transduction pathway by regulating the transcription of its own genes or membrane transport factors during oxidative stress to adjust its structure and function to adapt to stress; this process is called the Golgi stress response. When the Golgi stress is too intense, apoptosis is triggered (Li et al., 2016; Ignashkova et al., 2017). GM130 is a peripheral membrane protein strongly attached to the Golgi membrane and involved in the control of glycosylation, cell cycle progression, and higher-order cell functions such as cell polarization and directed cell migration. It is noteworthy that GM130 plays an important role in maintaining the Golgi structure (Nakamura, 2010). In addition, GM130 can regulate the configuration of F-actin, which is important for maintaining endothelial cell morphology and barrier function (Peng et al., 2011; Wang et al., 2015). Consistent with these observations, we previously found that the GA structure and function are closely related to the structure and function of the BBB (Deng et al., 2018). Interestingly, studies have shown that in the process of HSV-1 neuronal infection, fragmentation of the GA also occurred (Martin et al., 2017). However, the potentially deleterious effects that structural and functional changes of the GA may have on the BBB during HSV-1 infection have not been evaluated.

Many reports have demonstrated that viral infections can trigger apoptosis, a programmed cell death that plays an important role in viral pathogenesis and host antiviral response (Zhou et al., 2017). DeBiasi et al. (2002) found apoptotic neurons and glia in brain tissue sections of patients with acute HSE, indicating that HSV-1 infection can directly cause apoptosis of the BBB components. In addition, cleaved-caspase 3 is normally activated during HSV-1-triggered apoptosis (DeBiasi et al., 2002). Cleaved-caspase 3 is a vital effector caspase during the apoptotic process that chiefly cleaves cellular substrates important for maintaining the structural and biochemical integrity of the cell (Nguyen and Blaho, 2007). Bojarski et al. (2004) found that cleaved-caspase 3 can cleave occludin during apoptosis, resulting in the downregulation of occluding. These observations suggest that it is worth exploring the relationship between the GA, apoptosis, and the BBB during HSV-1 infection. The aim of the present study was to investigate the specific mechanism by which HSV-1 disrupts the BBB and whether Golgi stress plays a role in this process.

## Materials and Methods

### Biosafety Methodology

Our laboratories are equipped with biosafety equipment and containment barriers recommended by the World Health Organization (WHO) for type II risk organisms. HSV-1 propagation and infection experiments were carried out in the level 2 biosafety chamber provided by the Wuhan Institute of Virology. Moreover, we followed the necessary procedures for the disposal of biological elements and chemical reagents, according to the Biosafety Regulations from the Chinese Academy of Sciences.

### Cells and Cell Culture

Mouse brain endothelial cells (Bend.3) were purchased from Shanghai Zhong Qiao Xin Zhou Biotechnology company (ZQ-0090). African green monkey kidney cells (Vero) were purchased from ATCC (CRL-1586). These cells were cultured in Dulbecco’s modified Eagle medium (DMEM; Life Technologies; catalog number 12900017) supplemented with 10% fetal bovine serum (FBS; Life Technologies; catalog number 10099-141) and penicillin-streptomycin (100 U/ml and 100 μg/ml, respectively; Life Technologies; catalog number 15140122) at 37°C in a moist atmosphere containing 5% CO_2_.

### Viruses and Infections

A recombinant HSV-1 H129 stain created in the Luo laboratory in which the UL22-UL23 region was replaced by green fluorescent protein (GFP) regulated by the SV40 promoter was used in this study. HSV-1 was propagated and titrated using Vero cells as described previously (Zeng et al., 2017). Cells were synchronized by serum starvation prior to infection and then infected with HSV-1 for 2 h in DMEM containing 10% FBS, at a multiplicity of infection (MOI) of 3. Following infection, the medium was removed and replaced with fresh DMEM. Infected cells were collected at specific times as indicated in the figures. For inhibiting HSV-1-triggered apoptosis in Bend.3 cells, the cells were incubated with the pan-caspase inhibitor Z-VAD-fmk (100 μM; Selleck, USA) at 12 hours post-infection (hpi) and maintained during HSV-1 infection.

### Quantitation of Virus Replication

Bend.3 cells were seeded on 6-well (4 × 10^5^ cells/well) plates and then infected with HSV-1 H129-G4 at an MOI of 3. After 2 h of incubation, the medium was removed, the cells were rinsed with phosphate-buffered saline (PBS), and fresh DMEM was added. Supernatant samples were harvested at the indicated times and stored at −80°C until they were assayed. Infectious virus titers were identified by a plaque formation assay. Briefly, Vero cells were seeded into 24-well plates at a density of 2 × 10^6^ cells per plate. On the following day, the cells were infected with 200 μl of the supernatant sample after 10-fold serial dilution. After adsorption for 3 h, DMEM with a 0.5% agarose overlay was added to each well. Plaques were counted at 2–3 dpi, and an average titer was determined from three independent experiments.

### RNAi-Mediated GM130 Silencing

Cells were seeded 18–24 h prior to transfection to allow the monolayer cell density to reach 70–80% confluency at the time of transfection. Cells were transfected with small interfering RNA (siRNA; siCtrl, GenePharma), GM130 siRNA duplex 01 (siGM130-1, GenePharma) or GM130siRNA duplex 02 (siGM130-2, GenePharma). Transfection was performed with PepMute (SignaGen) according to the manufacturer’s instructions. Cells were harvested for lysate preparation or immunofluorescence 72 h after transfection.

### Cell Viability

Cell viability was detected by the 3-(4,5-dimethylthiazol-2-yl)-2,5-diphenyltetrazolium bromide (MTT) assay (Sigma-Aldrich) according to the manufacturer’s instructions. Briefly, the cells were seeded at 5 × 10^3^ cells/well in 96-well plates. On the following day, the cells were transfected with siCtrl, siGM130-1, or siGM130-2 and incubated for 72 h. The medium was then replaced with DMEM containing 0.5% MTT without phenol red. After incubation for 4 h at 37°C, the medium was discarded and replaced with 150 μl dimethyl sulfoxide (DMSO). The plate was shaken gently and the absorbance at 570 nm was measured with an Epoch microplate spectrophotometer (BioTek Instruments, Winooski, VT, USA).

### Plasmid Construction and Transfection

The mouse GM130-coding sequence was PCR amplified from cDNA prepared by reverse transcription (RT) of RNA extracted from Bend.3 cells and then cloned into the pcDNA3.1(+) plasmid to produce the GM130 expression construct pcDNA3.1-GM130. The GM130 PCR primers were as follows: 5′-ccaagctggctagcgctagcATGTGGCCCCCCCGCTTCCC-3′ and 5′-gtttaaacgggccctctagaTTATACAACCATGATCTTCA-3′. Uppercase letters represent sequences that match the template. Seamless clone was conducted by ClonExpress II One Step Cloning Kit (Vazyme, C112) with the manufacturer’s instructions. Transfections were carried out using Lipofectamine 3000 (Invitrogen; catalog number: L3000150) according to the manufacturer’s instructions. At 12 h post-transfection, Bend.3 cells were infected with HSV-1 for 36 h.

### Quantitative Reverse Transcription-PCR (qRT-PCR)

Bend.3 cells were cultured, infected at an MOI of 3, and harvested at the indicated times post-infection. A total of 1 × 10^6^ cells were used to obtain total RNA using the RNAiso Plus reagent (Takara; catalog number 9109), and residual DNA was removed with 10 U of recombinant DNase I (Takara; catalog number 2270A). One microgram of each RNA sample was reverse transcribed using RevertAid H Minus first-strand cDNA synthesis kit (Fermentas; catalog number K1631) with random primers. RT reaction products were quantified by a real-time thermocycler (Bio-Rad; Connect) with the iQ SYBR Green Supermix (Bio-Rad) in a 20 μl reaction mixture for 40 PCR cycles as described previously (Fu et al., 2015; Li et al., 2015; Yang et al., 2018). The PCR primers were as follows: GM130: 5′-ACGCAGAGAACCTGAAAGGA-3′ and 5′-GCCTCCAGCTCTTGTACCTG-3′ Claudin 5: 5′-GGCACTCTTTGTTACCTTGA-3′ and 5′-GGCATAAAGCGCTCCTCCCG-3′ Occludin: 5′-TACTGGTCTCTACGTGGATCAAT-3′ and 5′-TTCTTCGGGTTTTCACAGCAA-3′ GAPDH: 5′-TGGCCTTCCGTGTTCCTAC-3′ and 5′-GAGTTGCTGTTGAAGTCGCA-3′.

### Immunoblotting

For protein analysis, cells were harvested and lysed in RIPA buffer (Beyotime; catalog number P0013) supplemented with protease inhibitor cocktail (Roche; catalog number 04693159001) and the protein concentration was determined by the Bradford assay (Bio-Rad; catalog number 500-0205). Equal amounts of protein were loaded and separated by SDS-PAGE and transferred to polyvinylidene difluoride membranes (Millipore; catalog number ISEQ00010), which were then probed with antibodies as previously described (Yang et al., 2018). Primary antibodies used included anti-GM130 (BD Biosciences Cat# 610822 Lot# RRID:AB_398141), anti-P115 (Proteintech Cat# 13509-1-AP Lot# RRID:AB_2257094), anti-cleaved caspase-3 (Cell Signaling Technology Cat# 9661 Lot# RRID:AB_2341188), anti-claudin 5 (Thermo Fisher Scientific Cat# 34-1600 Lot# RRID:AB_2533157), anti-occludin (Thermo Fisher Scientific Cat# 33-1500 Lot# RRID:AB_2533101), and anti-GAPDH (Proteintech Cat# 10494-1-AP Lot# RRID:AB_2263076). Secondary antibodies used were horseradish peroxidase-conjugated goat anti-mouse IgG (Jackson ImmunoResearch Labs Cat# 115-035-003 Lot# RRID: AB_10015289) and donkey anti-rabbit IgG (Jackson ImmunoResearch Labs Cat# 111-035-003 Lot# RRID: AB_2313567). The bands were detected using SuperSignal West Femto chemiluminescent substrate (Life Technologies; catalog number 34095) and the FluorChem HD2 system (Alpha Innotech). The resulting images were analyzed by densitometry using ImageJ software (National Institutes of Health).

#### Immunofluorescence Analysis

Non-infected Bend.3 (Mock) and HSV-1 infected Bend.3 cells [treated or untreated with pan-caspase inhibitor Z-VAD-fmk (Selleck; S7023)] were fixed in 4% paraformaldehyde for 30 min and washed in 1× PBS. The cells were then incubated with primary antibodies against GM130 (BD Biosciences Cat# 610822 Lot# RRID:AB_398141), P115 (Proteintech Cat# 13509-1-AP Lot# RRID:AB_2257094), cleaved caspase 3 (Cell Signaling Technology Cat# 9661 Lot# RRID:AB_2341188), and claudin 5 (Thermo Fisher Scientific Cat# 34-1600 Lot# RRID:AB_2533157) overnight at 4°C. Subsequently, the cells were incubated with Alexa Fluor 488-conjugated (Molecular Probes Cat# A-21202 also A21202 Lot# RRID: AB_141607), Alexa Fluor 594-conjugated (Molecular Probes Cat# A-21207 also A21207 Lot# RRID: AB_141637), and Alexa Fluor 647-conjugated (Molecular Probes Cat# A-21240 also A21240 Lot# RRID: AB_141658) secondary antibodies. Nuclei were stained with DAPI (4,6-diamidino-2-phenylindole; catalog number D9542; Sigma-Aldrich). Fluorescence images were acquired using an UltraVIEW VoX spinning disk laser confocal scanning microscope (PerkinElmer). At least five microscopic fields of each sample were captured. We defined the maximum distance between the Golgi marker (GM130) and the nucleus as a parameter of GA dispersion. Immunofluorescence images (30 cells for each condition) acquired from mock-infected or HSV-1 infected Bend.3 cells in the absence or presence of Z-VAD-fmk were analyzed in each condition. Each image was calibrated (pixels/μm) with ImageJ software (National Institutes of Health).

#### Transmission Electron Microscopy

Cells were infected with HSV-1 (MOI = 3) and harvested at the times post-infection as indicated in the figures. Cells were fixed for 1 h at 25° in 2.5% glutaraldehyde and then incubated with 1% osmium tetroxide (OsO_4_), dehydrated in a gradient of ethanol (30–100%) and embedded with an Embed 812 kit (Electron Microscopy Sciences, Fort Washington, PA, USA). Ultrathin sections (60–80 nm) were obtained, mounted on copper grids (200 mesh), and stained with 2% (wt/vol) phosphotungstic acid (PTA; pH 6.8). Observations of the grids were made using a Tecnai transmission electron microscope (FEI) operated at 200 kV. Additionally, in order to reflect the extent of GA damage, we measured the Golgi cisternae lumen width (μm) in mock- and HSV-1-infected cells transfected with pcDNA3.1 or pcDNA3.1-GM130. We quantified the maximum length of GA cisternae (designated as maximum luminal width) by measuring the cisternae diameters assuming circular structures. Thirty cells for each condition and 3–5 GA cisternae for each cell were analyzed by electron microscopy. Each image was measured with ImageJ software (National Institutes of Health; Martin et al., 2017).

#### Measurement of Apoptosis

Quantitation of apoptotic cells under HSV-1 infection was obtained using the Annexin V-FITC detection kit (Beyotime) according to the manufacturer’s protocol. Briefly, Bend.3 cells were collected and resuspended with 195 μl Annexin V-FITC Binding Buffer. Next, 5 μl Annexin V-FITC and 10 μl propidium iodide were added to each group and the cells were incubated at 25° for 10–20 min in dark. The percentage of apoptotic cells was detected using a flow cytometer (BD Biosciences). FlowJo software was used for flow cytometry analysis.

#### Measurement of Transendothelial Electrical Resistance (TEER)

Endothelial permeability can be assessed by measuring TEER using a Millicell ERS Voltohmmeter (Millipore, Billerica, MA, USA). The TEER of Bend.3 monolayers were obtained by subtracting the resistance of cell-free inserts and multiplying the resulting value by the membrane surface area of inserts. The values were expressed as Ω•cm^2^.

#### Statistical Analysis

All the data are representative of at least three independent experiments and expressed as mean ± SD. The results were analyzed by the Student’s *t*-test for comparison of two experimental groups at one time-point, and *p* < 0.05 was considered statistically significant. Two-way ANOVA was used for the comparison of two experimental groups at multiple time points, and *p* < 0.05 was considered statistically significant. The bar graphs were generated with GraphPad Prism software package (version 5.01; IBM).

## Results

### HSV-1 Infection Downregulates GM130, Disrupts the GA, and Induces Apoptosis

**Figure 1:**
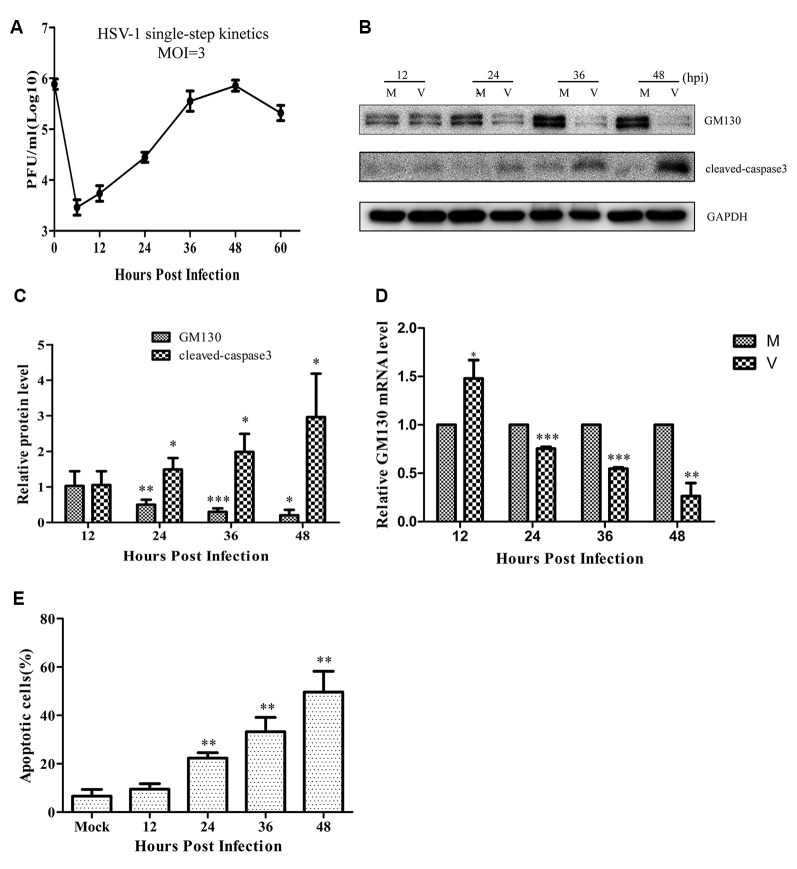
Herpes simplex virus-1 (HSV)-1 infection downregulates GM130 and induces apoptosis in Bend.3 cells. **(A)** Growth kinetics of HSV-1 infection in Bend.3 cells. Bend.3 cells were infected with HSV-1 at an multiplicity of infection (MOI) of 3. Supernatant samples were collected at the indicated times post-infection. Viral titer was measured with a plaque formation assay in triplicate. Bend.3 cells were either mock-infected (M) or infected with HSV-1 (V) at an MOI of 3 and harvested at the times indicated (in hours). **(B)** GM130 and cleaved-caspase 3 protein levels were detected by immunoblotting at the times indicated and are expressed as fold changes relative to mock-infected cells after normalization to GAPDH levels at each time point. GAPDH is a loading control. Representative images are shown from three independent experiments. Quantification of **(B)** is shown in **(C)**. **(D)** The mRNA levels of GM130 were downregulated in HSV-1-infected cells. GM130 mRNA levels were analyzed by qRT-PCR and are normalized to the levels in mock-infected cells to provide fold changes after normalization to GAPDH levels. GAPDH serves as an internal control. **(E)** The extent of apoptosis in Bend.3 cells induced by HSV-1 infection. Apoptotic cells were detected by Annexin-V binding and PI staining. Each experiment was performed independently three times. Data are presented as mean ± SD and were analyzed by the Student’s *t*-test; *P < 0.05; **P < 0.01; ***P < 0.001.

Since the GA is a significant organelle for both the host and the virus, we wanted to investigate whether brain microvascular endothelial cells infected by HSV-1 could trigger changes in the GA. At first, the replication and propagation properties of the recombinant HSV-1 129 strain in Bend.3 cells were determined by the one-step growth curve. The result showed that the viral titer reached a peak at 48 hpi with a maximum titer of 9 × 10^5^ PFU/ml, and then the viral load showed a gradual decrease (Figure 1A).

As an important structural protein of the GA, GM130 maintains the structure of the GA with P115, Giantin, GRASP65, Rab GTPase, and other Golgi-related proteins. Previous studies have demonstrated that GM130 can regulate the integrity of the GA, hence we assessed whether GM130 expression is altered during infection of endothelial cells. As shown in Figures 1B–D, after 24 h of infection, both the protein and mRNA levels of GM130 began to decrease (*p* < 0.05). As the infection progressed, the protein and mRNA levels of GM130 continued to decrease. All original images of immunoblotting in the article can be found in the Supplementary Datasheet 1.

**Figure 2:**
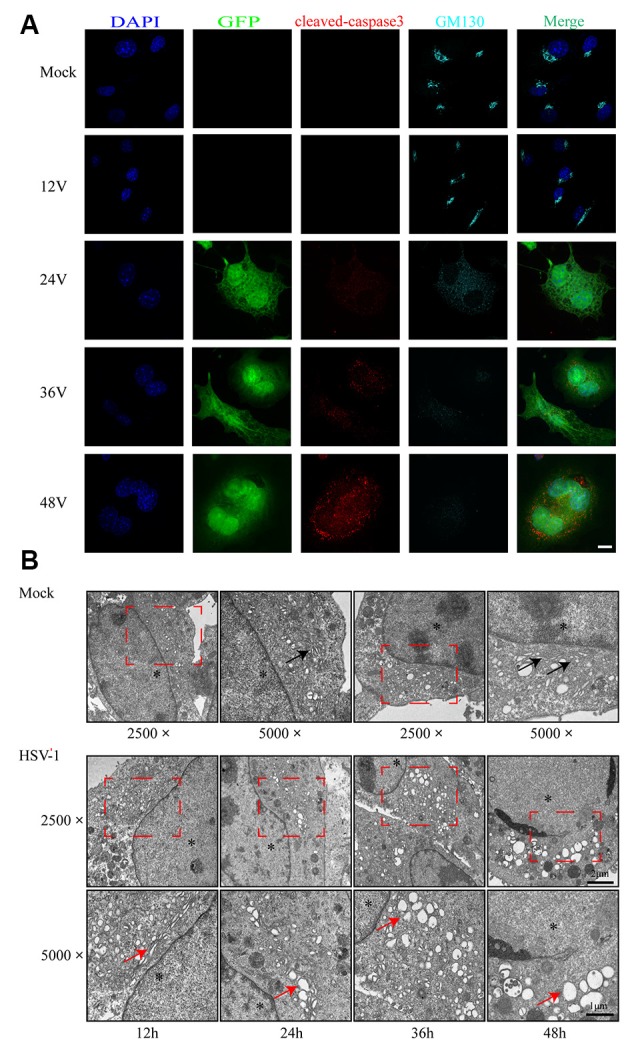
Golgi apparatus (GA) disruption and cleaved-caspase 3 activations in Bend.3 cells infected with HSV-1. Bend.3 cells were mock-infected (mock) or infected with HSV-1 (V) at an MOI of 3 and then harvested at 12 hours post-infection (hpi), 24 hpi, 36 hpi, and 48 hpi. **(A)** GA disruption and cleaved-caspase 3 activations were detected by immunofluorescence analysis. Mock-infected or HSV-1 infected cells were fixed, permeabilized, and incubated with antibodies to GM130 and cleaved-caspase 3, followed by incubation with Alexa Fluor 594-conjugated IgG (red channel) and Alexa Fluor 647-conjugated IgG (cyan channel). Infected cells expressed green fluorescent protein (GFP; green channel). DAPI was used to stain nuclei (blue channel). Merged channels generated the fifth image shown in each row. Scale bar, 10 μm. **(B)** GA disruption was determined by transmission electron microscopy. GA regions (red box) were captured at a magnification of 2,500× in mock-infected (upper) and HSV-1 infected (lower) cells. Scale bar, 2 μm. Highly ordered Golgi stacks are observed in mock-infected cells at a magnification of 5,000× (black arrowheads). The ultrastructure of the GA in HSV-1-infected cells gradually varied from organized stacks to discontinued stacks and dilated-elongated Golgi cisternae (red arrowheads). Representative images are shown from three independent experiments. The asterisk indicates the nucleus. Scale bar, 1 μm.

As shown in Figure 2A, infected cells were identified by the detection of GFP and detected 24 h after infection. We observed the structural changes of GA by immunofluorescence analysis and electron microscopy. Figure 2A shows that as the infection time increased, the GA pattern indicated by GM130 immunofluorescence in infected cells was gradually altered in comparison to mock-infected cells. After infection, the GM130 exhibited a dispersed and fragmented distribution throughout the cytoplasm, unlike the mock-infected cells where it accumulated in the perinuclear in a crescent-shaped pattern.

We also observed the alteration in the ultrastructure of the GA by transmission electron microscopy during the course of HSV-1 infection. We first observed at a magnification of 2,500× and then increased the magnification of the area of interest to 5,000×. As shown in Figure 2B, the GA is initially highly ordered, appearing as ribbon-like stacks in a vesicular structure. The GA begins to swell at 24 h hpi, shows a large bubble shape at 48 hpi, and finally, some fragments are visible. The timing of the Golgi disruption as shown by immunofluorescence and electron microscopy was consistent with that of the GM130 alteration, revealing a correlation between GM130 and GA fragmentation upon HSV-1 infection.

Cells infected with the virus will die in many ways, with apoptosis being the most common. To determine whether the cells were apoptotic, we performed flow cytometry and immunoblot analyses. The flow cytometry results showed no difference in the apoptosis rate between the infected and control groups at 12 hpi (Figure 1E). However, the apoptosis rates began to differ significantly at 24 hpi. As the infection time increased, the apoptosis rate gradually increased, reaching 49.2% at 48 hpi. Cleaved-caspase 3 levels increased after HSV-1 infection (Figures 1B,C), and was distributed throughout the cytoplasm in infected cells (Figure 2A). These results indicate that HSV-1 infection triggers apoptosis, thereby activating cleaved-caspase 3, a downstream effector caspase.

### HSV-1 Infection Disrupts Endothelial Cell Barrier Structure and Function

**Figure 3:**
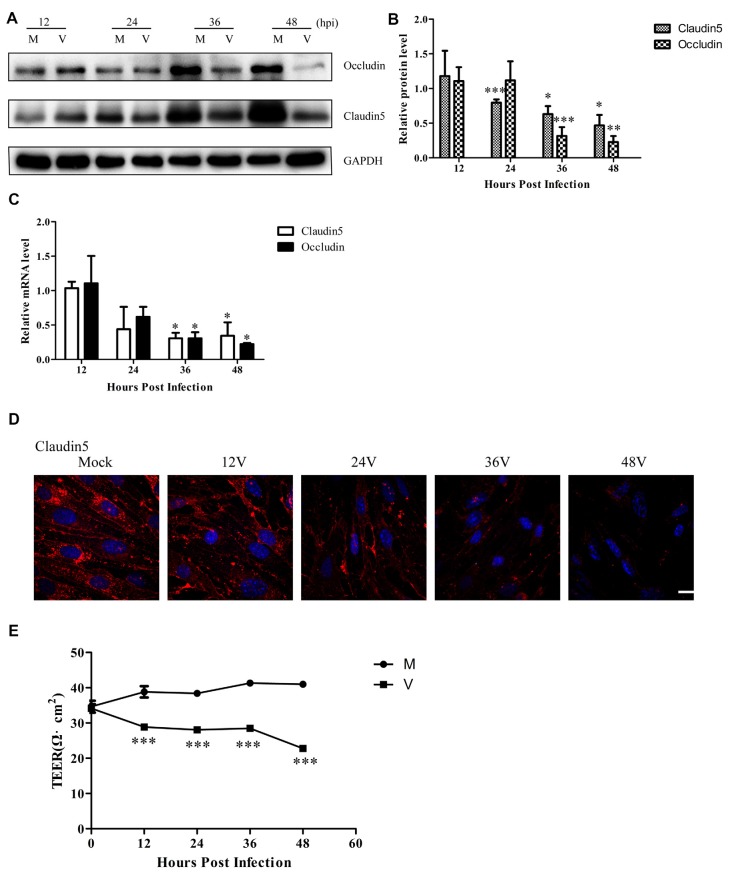
Endothelial cell barrier structure and function disruption after HSV-1 infection. Bend.3 cells were mock-infected (mock) or infected with HSV-1 (V) at an MOI of 3 and harvested at the times indicated. **(A)** The protein levels of occludin and claudin 5 were downregulated by HSV-1 infection. Occludin and claudin 5 protein levels were determined by immunoblotting and are expressed as fold differences relative to mock-infected cells after normalization to GAPDH levels. GAPDH serves as a loading control. Quantification of **(A)** is shown in **(B)**. Data are shown as mean ± SD from three independent experiments and were analyzed by the Student’s *t*-test; *P < 0.05; **P < 0.01; ***P < 0.001. **(C)** The mRNA levels of occludin and claudin 5 were downregulated by HSV-1 infection. Occludin and claudin 5 mRNA levels were quantified by qRT-PCR and presented as fold differences relative to the levels in mock-infected cells after normalization to GAPDH levels. GAPDH serves as an internal control. Each experiment was performed independently three times. Data are presented as mean ± SD and were analyzed by the Student’s *t*-test; *P < 0.05. **(D)** Claudin 5 (red) was detected by immunofluorescence analysis and nuclei were stained with DAPI (blue). Representative images are shown from three independent experiments. Scale bar, 10 μm. **(E)** Bend.3 monolayer permeability was assessed by TEER measurements. The results show that the barrier function of Bend.3 was disrupted with increasing infection times. Data shown are presented as mean ± SD from three independent experiments and were analyzed by two-way ANOVA. ***P < 0.001.

Previous experiments have shown that cleaved-caspase 3 contributes to claudin 5 and zonula occludens-1 tight junction disruption in rapid anoxic neurovascular unit damage (Zehendner et al., 2011). In the present study, we found cleaved-caspase 3 activation in Bend.3 cells infected with HSV-1. Thus, we next examined whether HSV-1 infection affects the expression of the tight junction proteins, occludin and claudin 5. Immunoblotting analysis showed that occludin and claudin 5 were downregulated 36 h and 24 h after infection, respectively (Figures 3A,B). Concurrently, we found a decrease in the transcription levels of occludin and claudin 5 (Figure 3C). Immunofluorescence analysis (Figure 3D) revealed that claudin 5 is clearly and linearly distributed across the cell membrane prior to HSV-1 infection. After HSV-1 infection, the claudin 5 pattern becomes discontinuous, appearing almost as a dot-like structure or disappearing altogether. Since these observations may reflect changes in the endothelial cell barrier structure, we further evaluated the endothelial cell barrier function. Measurements of TEER that indicate endothelial monolayer permeability changes confirmed that HSV-1 infection results in a time-dependent decrease in TEER (Figure 3E). Taken together, these observations strongly suggest that HSV-1 infection disrupts the endothelial cell barrier structure and function in Bend.3 cells.

### Knockdown of GM130 Disrupts the GA, Induces Apoptosis, and Downregulates Protein Levels of Occludin and Claudin 5

**Figure 4:**
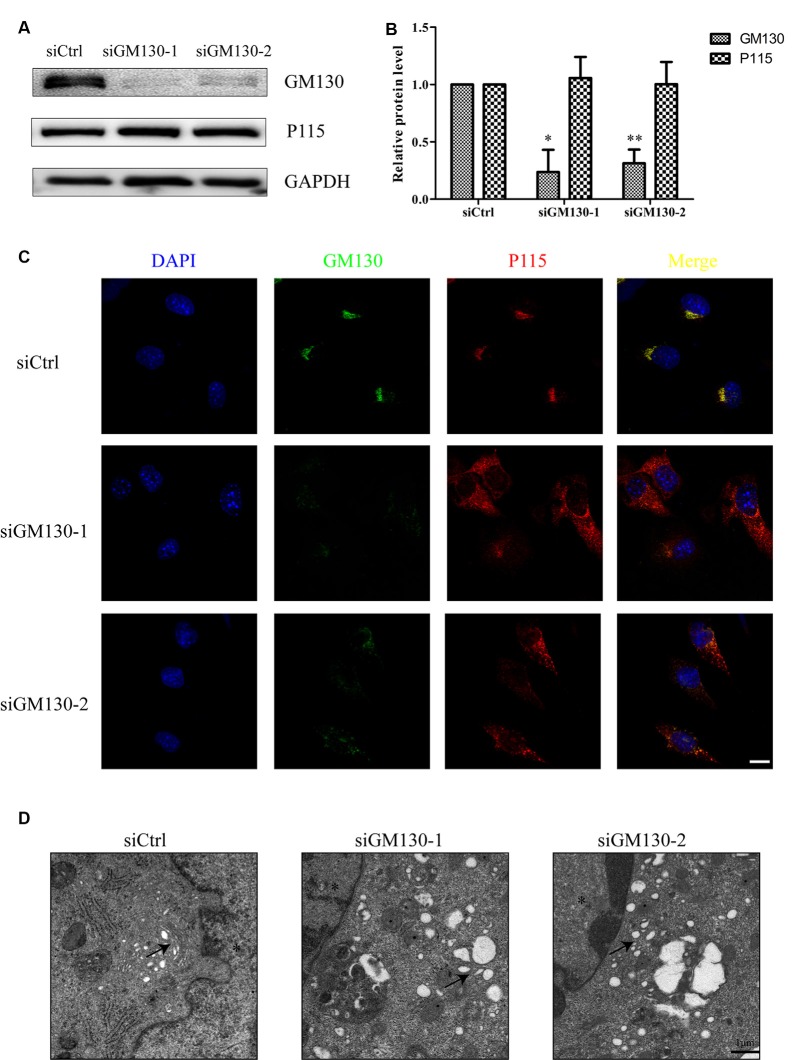
GA fragmentation after knockdown of GM130 in Bend.3 cells. Bend.3 cells were transfected with a negative control small interfering RNA (siCtrl), and two GM130-specific siRNA (siGM130-1, siGM130-2) and harvested at 72 h post-transfection. **(A)** P115 was not affected by GM130 knockdown. GM130 and P115 protein levels were determined by immunoblotting and presented as fold differences relative to the levels in siCtrl-transfected cells after normalization to GAPDH levels. GAPDH serves as a loading control. Quantification of **(A)** is shown in **(B)**. Data are shown as mean ± SD from three independent experiments and were analyzed by the Student’s *t*-test; *P < 0.05; **P < 0.01; **(C)** GA fragmentation was detected by immunofluorescence assay; transfected cells were immunostained for GM130 (Golgi marker protein, green) and P115 (Golgi marker protein, red). Nuclei were visualized with DAPI. Scale bar, 10 μm. **(D)** The morphological disruption of the GA was determined by transmission electron microscopy. Representative electron micrographs of Golgi stacks were captured in the negative control group and GM130 knockdown group at 5,000× magnification. Highly ordered and compact Golgi stacks became disordered and dilated-elongated after knockdown (black arrowheads). Representative images are shown from three independent experiments. The asterisk indicates the nucleus. Scale bar, 1 μm.

We have shown that the GA and GM130 are significantly altered after HSV-1 infection. To further determine the role of the GA and GM130 during HSV-1 infection, we knocked down GM130 in Bend.3 cells using small interfering RNA (siRNA). We confirmed that there were no significant differences in cell viability between cells transfected with the negative control (siCtrl) and GM130-specific (siGM130-1 or siGM130-2) siRNA (Supplementary Datasheet 2). P115, another Golgi marker, was not affected on the protein level by GM130 knockdown (Figures 4A,B). Next, we used immunofluorescence analysis of GM130 and P115 to assess the GA structure (Figure 4C). The GA structure varied from a compacted, perinuclear distribution to a dispersed distribution throughout the cytoplasm. The morphological disruption of the GA was further examined by transmission electron microscopy. Representative electron micrographs of Golgi stacks captured in the negative control group (siCtrl) and GM130 knockdown groups (siGM130-1 and siGM130-2) showed that the highly ordered and compact Golgi stacks became disordered and dilated/elongated after GM130 knockdown (Figure 4D). These results indicate that GM130 is required for maintaining the GA structure.

**Figure 5:**
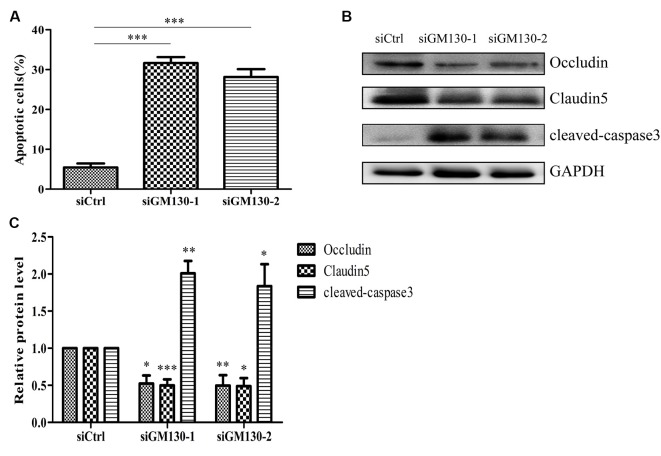
Knockdown of GM130 induces apoptosis and downregulates protein levels of occludin and claudin 5 in Bend.3 cells. Bend.3 cells were transfected with a negative control small interfering RNA (siCtrl) and two GM130-specific siRNA (siGM130-1, siGM130-2) and harvested at 72 h post-transfection. **(A)** Apoptotic cells were detected by flow cytometry after GM130 knockdown. **(B)** Cleaved-caspase 3, occludin, and claudin 5 protein levels were determined by immunoblotting and expressed as fold changes relative to siCtrl-transfected cells after normalization to GAPDH levels. GAPDH serves as a loading control. Quantification of **(B)** is shown in **(C)**. Data are shown as mean ± SD from three independent experiments and were analyzed by the Student’s *t*-test; *P < 0.05; **P < 0.01; ***P < 0.001.

Next, we used flow cytometry analysis and immunoblot detection of cleaved-caspase 3 to detect cell apoptosis. The significant increase in the percentage of apoptotic cells (Figure 5A) and protein level of cleaved caspase 3 (Figures 5B,C) in the GM130 knockdown groups indicate that loss of GM130 may play a role in apoptosis caused by HSV-1 infection. Moreover, we observed that knockdown of GM130 downregulated the protein levels of occludin and claudin 5 (Figures 5B,C). These results indicate that GM130 may be involved in apoptosis and downregulation of occludin and claudin 5 in HSV-1-infected Bend.3 cells.

### Overexpression of GM130 Ameliorates Apoptosis, Restores the Protein Levels of Occludin and Claudin 5, and Reduces Golgi Fragmentation During HSV-1 Infection

**Figure 6:**
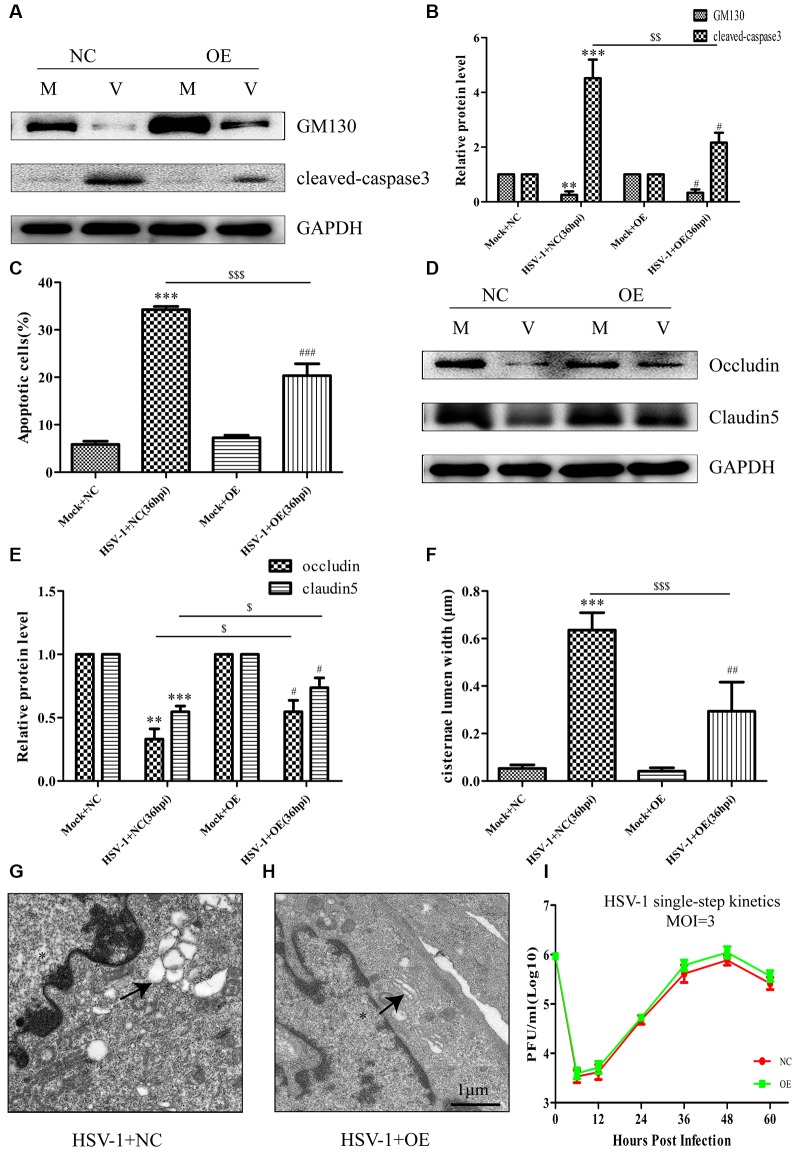
Overexpression of GM130 ameliorates apoptosis, restores the protein levels of occludin and claudin 5, and reduces Golgi fragmentation during HSV-1 infection. Bend.3 cells were transfected with either a negative control plasmid pcDNA3.1 (NC) or GM130 expression plasmid pcDNA3.1-GM130 (OE). Twelve hours after transfection, cells were infected with HSV-1 for 36 h and then harvested. **(A)** GM130 and cleaved-caspase 3 protein levels were determined by immunoblotting. GM130 and cleaved-caspase 3 protein levels in HSV-1-infected cells transfected with pcDNA3.1 or pcDNA3.1-GM130 were normalized to the levels in mock-infected cells transfected with pcDNA3.1 or pcDNA3.1-GM130, respectively, to provide fold changes after normalization to GAPDH levels. GAPDH is a loading control. Quantification of **(A)** is shown in **(B)**. Data are shown as mean ± SD from three independent experiments and were analyzed by the Student’s *t*-test; **P < 0.01; ***P < 0.001 vs. Mock + NC; ^$$^*P* < 0.01 vs. HSV-1 + NC; ^#^*P* < 0.05 vs. Mock + OE. **(C)** Apoptotic cells were quantified by flow cytometry. Data shown are presented as mean ± SD from three independent experiments and were analyzed by the Student’s *t*-test; ***P < 0.001 vs. Mock + NC; ^$$$^*P* < 0.001 vs. HSV-1 + NC; ^###^*P* < 0.001 vs. Mock + OE. **(D)** Occludin and claudin 5 protein levels were determined by immunoblotting. Occludin and claudin 5 protein levels in HSV-1-infected cells transfected with pcDNA3.1 or pcDNA3.1-GM130 were normalized to the levels in mock-infected cells transfected with pcDNA3.1 or pcDNA3.1-GM130, respectively, to provide fold changes after normalization to GAPDH levels. GAPDH is a loading control. Quantification of **(D)** is shown in **(E)**. Data are shown as mean ± SD from three independent experiments and were analyzed by the Student’s *t*-test; **P < 0.01; ***P < 0.001 vs. Mock + NC; ^$^*P* < 0.05 vs. HSV-1 + NC; ^#^*P* < 0.05 vs. Mock + OE. **(F)** Quantitation of Golgi cisternae lumen (μm) in mock- and HSV-1-infected cells transfected with pcDNA3.1 or pcDNA3.1-GM130. Data represents the average maximum luminal width of Golgi cisternae from three independent experiments. Data are shown as mean ± SD and were analyzed by the Student’s *t*-test; ***P < 0.001 vs. Mock + NC; ^$$$^*P* < 0.001 vs. HSV-1 + NC; ^##^*P* < 0.01 vs. Mock + OE. Representative electron micrographs of Golgi cisternae in HSV-1-infected cells transfected with pcDNA3.1 **(G)** and HSV-1-infected cells transfected with pcDNA3.1-GM130 at 36 hpi **(H)**. Representative images are shown from three independent experiments. The black arrowheads indicate Golgi cisternae. The asterisks indicate the nucleus. Scale bar, 1 μm. **(I)** Growth kinetics of viruses. Twelve hours after transfection, Bend.3 cells transfected with pcDNA3.1 or pcDNA3.1-GM130 were infected with HSV-1 at an MOI of 3. The supernatant was collected at the indicated times post-infection, and viral titer was measured with a plaque formation assay in triplicate. Data shown are presented as mean ± SD from three independent experiments and were analyzed by two-way ANOVA.

To further confirm the involvement of GM130 in apoptosis and downregulation of occludin and claudin 5 after HSV-1 infection, Bend.3 cells were transfected with either the pcDNA3.1 empty vector or pcDNA3.1-GM130 plasmid. Twelve hours after transfection, the cells were infected with HSV-1 for 36 h. GM130 was clearly overexpressed in both mock- and HSV-1-infected Bend.3 cells (Figures 6A,B). Overexpression of GM130 partially inhibited the activation of cleaved-caspase 3 (Figures 6A,B). Correspondingly, Bend.3 cells transfected with pcDNA3.1-GM130 displayed a decrease in the number of apoptotic cells after HSV-1 infection compared with cells transfected with the empty vector (Figure 6C). We also observed that the protein levels of occludin and claudin 5 were partially restored by overexpressing GM130 in HSV-1-infected Bend.3 cells (Figures 6D,E). Furthermore, we detected GA ultrastructural changes after GM130 overexpression. As shown in Figures 6F–H, overexpression of GM130 improved the disordered and dilated Golgi cisterna caused by HSV-1 infection. Quantitation of the maximum luminal width of the Golgi cisternae (μm) revealed a significant decrease in this parameter. Overexpression of GM130 had no significant impact on infectious virus yields at the indicated times post-infection (Figure 6I). Taken together, these results strongly suggest that HSV-1 infection triggers GM130-mediated GA stress presenting as Golgi fragmentation, which is involved in apoptosis and downregulation of occludin and claudin 5 protein levels.

### GM130 Downregulation, GA Fragmentation, and Endothelial Cell Barrier Structure and Function Disruption During HSV-1 Infection-Induced Apoptosis Are Partially Caspase-Dependent

**Figure 7:**
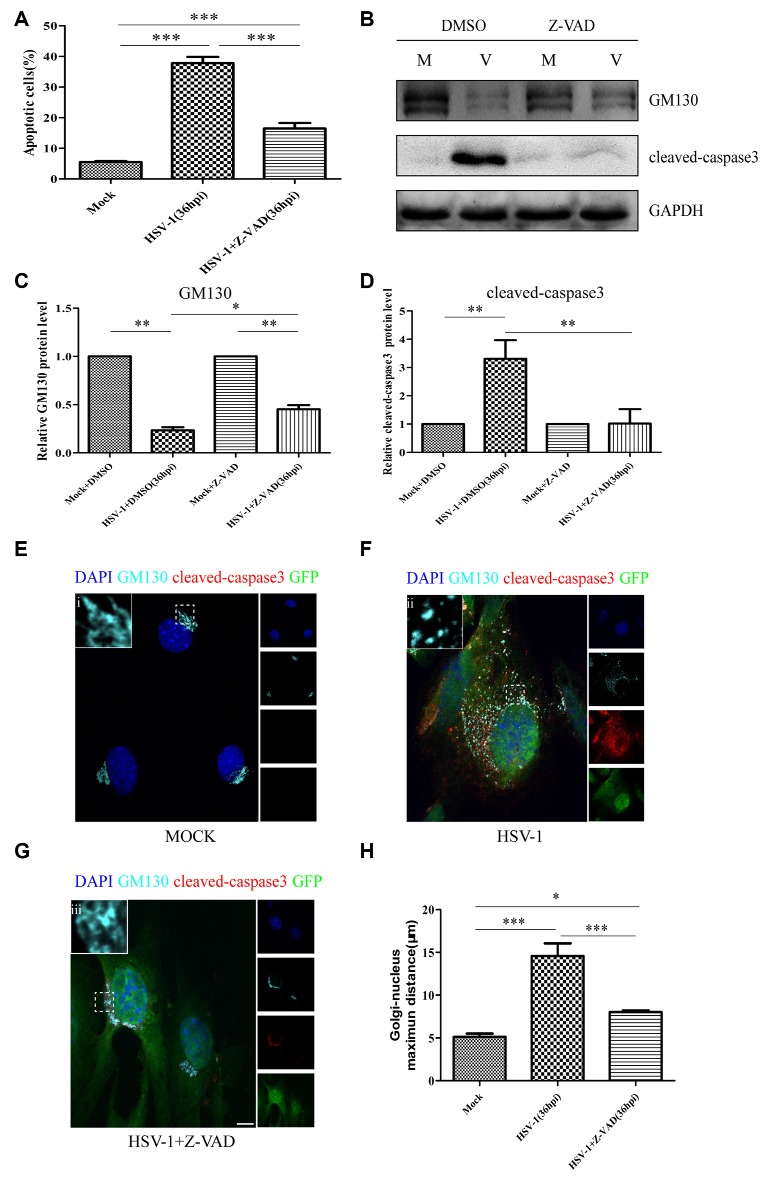
Z-VAD-fmk inhibits apoptosis, restores GM130 protein levels, and reduces GA dispersion during HSV-1 infection. At 12 hpi, mock-infected (Mock) and HSV-1-infected (V) cells were treated with either dimethyl sulfoxide (DMSO) or Z-VAD-fmk (100 μM) and then harvested at 36 hpi. **(A)** Apoptotic cells were quantified by flow cytometry and **(B)** GM130 and cleaved-caspase 3 protein levels were determined by immunoblotting in HSV-1-infected cells treated with DMSO or Z-VAD-fmk and expressed as fold changes relative to mock-infected cells treated with DMSO or Z-VAD-fmk, respectively, after normalization to GAPDH levels. GAPDH serves as a loading control. Quantification of **(B)** is shown in **(C,D)**. Data are shown as mean ± SD from three independent experiments and were analyzed by the Student’s *t*-test; *P < 0.05; **P < 0.01; cells that were mock-infected **(E)**, HSV-1-infected **(F)**, or HSV-1-infected with Z-VAD-fmk treatment **(G)** were immunostained for GM130 (Golgi marker protein, cyan), cleaved-caspase 3 (cell apoptosis marker, red), and GFP (viral infection marker, green). Nuclei were stained with DAPI. Panels **(E–G)** show boxes (i-iii) with higher magnification to indicate the GA structure. Scale bar, 1 μm. **(H)** Quantitation of the maximum distance of GM130 positive structures to the nucleus (μm) under the conditions described in Panels **(E–G)**. All experiments were repeated independently at least three times. Data are shown as mean ± SD and were analyzed by the Student’s *t*-test; *P < 0.05; **P < 0.01; ***P < 0.001.

Cellular apoptosis results in the activation of a family of cysteine proteases named caspases that act in a proteolytic cascade to cleave relevant proteins. Z-VAD-fmk is an irreversible general caspase inhibitor, which can inhibit apoptosis in some cases. Firstly, to determine whether the apoptosis induced by HSV-1 infection can be inhabited by Z-VAD-fmk, Bend.3 cells were infected with HSV-1 in the absence or presence of Z-VAD-fmk. Flow cytometry analysis showed that in the presence of Z-VAD-fmk, the apoptotic rate of HSV-1 infected cells was significantly lower than the untreated group (Figure 7A). Concurrently, the activation of cleaved-caspase 3 was completely inhibited by Z-VAD-fmk (Figures 7B,C). These results confirmed that Z-VAD-fmk can protect cells from apoptosis during HSV-1 infection.

**Figure 8:**
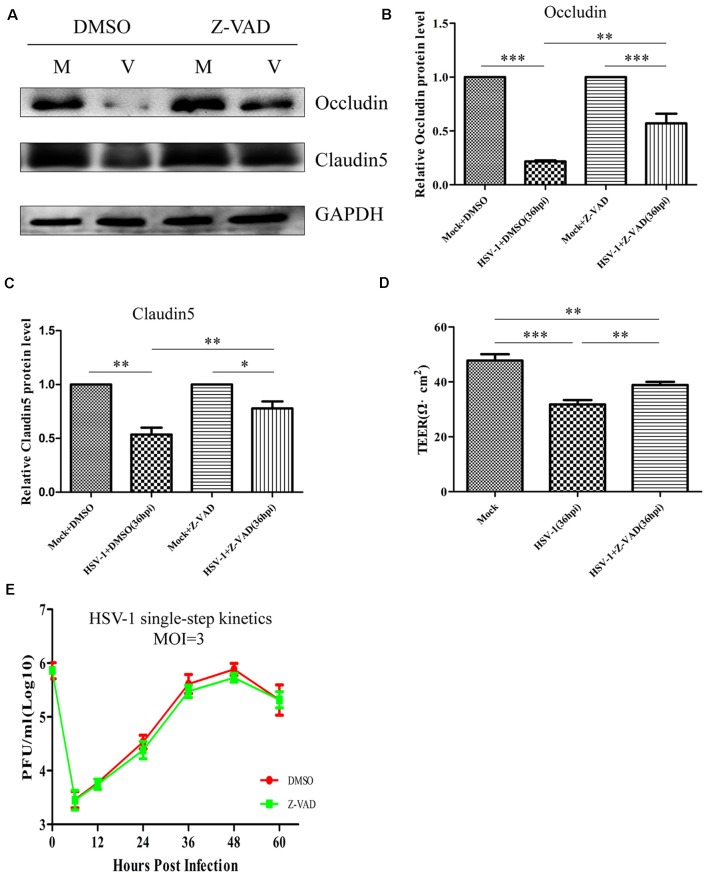
Z-VAD-fmk improves endothelial cell barrier structure and function during HSV-1 infection. At 12 hpi, mock-infected (Mock) and HSV-1-infected (V) Bend.3 cells were treated with DMSO or Z-VAD-fmk (100 μM) and then harvested at 36 hpi. **(A)** Occludin and claudin 5 protein levels were determined by immunoblotting in HSV-1-infected cells treated with DMSO or Z-VAD-fmk and expressed as fold changes relative to mock-infected cells treated with DMSO or Z-VAD-fmk, respectively, after normalization to GAPDH levels. GAPDH serves as a loading control. Quantification of **(A)** is shown in **(B,C)**. **(D)** Bend.3 barrier function was assayed by TEER measurements. The results show the barrier function of Bend.3 was improved by treatment with Z-VAD-fmk during HSV-1 infection. All data are presented as mean ± SD from three independent experiments and were analyzed by the Student’s *t*-test; *P < 0.05; **P < 0.01; ***P < 0.001. **(E)** Growth kinetics of viruses. Bend.3 cells were infected with HSV-1 at an MOI of 3. Supernatant samples from HSV-1-infected cells were collected at the indicated times post-infection. Prior to each sample collection, HSV-1-infected cells were preincubated with DMSO or Z-VAD-fmk (100 μM) for 24 h. Viral titer was measured with a plaque formation assay in triplicate. Data shown are presented as mean ± SD from three independent experiments and were analyzed by two-way ANOVA.

We next investigated whether the decreased GM130 protein level, GA fragmentation, and endothelial cell barrier structure and function disruption caused by HSV-1 infection were caspase-dependent. Bend.3 cells were infected with HSV-1 in the absence or presence of Z-VAD-fmk and the protein level of GM130 was assessed. Z-VAD-fmk treatment partially restored the protein level of GM130, demonstrating that the restoration is caspase-dependent (Figures 7B,D). Furthermore, to assess whether caspase activity plays a role in GA fragmentation, we examined the distribution of GM130 in the cells. We found that Z-VAD-fmk reduced the dispersed and fragmented distribution of GM130 (Figures 7E–G). Quantification of the distance of GM130-positive structures to the nucleus showed a significant reduction in this parameter at 36 hpi (Figure 7H). Finally, we observed that treatment with Z-VAD-fmk significantly increased the protein levels of occludin and claudin 5 (by 32% and 24%, respectively) at 36 hpi (Figures 8A–C). Correspondingly, the TEER was also improved (Figure 8D). Z-VAD-fmk had no significant impact on infectious virus yields at the indicated times post-infection (Figure 8E). Altogether, these results indicate that caspase activity is required to cleave GM130, trigger GA fragmentation, and disrupt endothelial cell barrier structure and function during apoptosis caused by HSV-1 infection in Bend.3 cells.

## Discussion

HSV-1 is one of the most common pathogens that infect the CNS. Despite the use of antiviral drugs, the mortality rate of HSE caused by HSV-1 remains as high as 20–30%, and more than half of surviving patients suffer varying degrees of neurological deficits (Saraya et al., 2016). There is robust evidence that the destruction of the BBB is an important pathological mechanism in the development of HSE (Buursma et al., 2005; Keep et al., 2008; Moretti et al., 2015; Liu et al., 2019b), but the underlying mechanism remains to be elucidated. Previous results from studies by our laboratory and others (Martin et al., 2017; Deng et al., 2018) motivated us to explore whether the GA plays a role in the destruction of the BBB in HSE.

Not only does the GA secrete and process proteins, but the evidence is accumulating that it is a sensor of stress signals in cell death pathways. In addition, recent experiments indicate that with increasing confluence of endothelial cells, cPLA relocation to the GA also increases, which facilitates the transport of occludin and claudin 5 through or from the Golgi, thereby controlling the integrity of endothelial cell-cell junctions (Regan-Klapisz et al., 2009). In the present study, we observed that after HSV-1 infection, the GA marker protein GM130 gradually exhibited a dispersed and fragmented distribution throughout the cytoplasm. GA fragmentation was further confirmed by transmission electron microscopy. Concurrent with the GA disruption, downregulation of occludin and claudin 5 and apoptosis of endothelial cells were observed. However, when the GA structure was restored by overexpressing GM130 in HSV-1-infected cells, the downregulation of occludin and claudin 5 and apoptosis of endothelial cells were ameliorated to a certain extent. Our results are partially consistent with the observations by Nakagomi that inhibition of GA fragmentation could inhibit or delay neuronal apoptosis triggered by multiple apoptotic insults (Nakagomi et al., 2008). Moreover, occludin, claudin 5, and endothelial cells are important components of the BBB. Thus, our findings reveal that HSV-1 infection triggers the Golgi stress response, which mainly presents as GA fragmentation, and these changes in Golgi morphology and function affect the BBB.

GM130 is an important structural protein of the GA, which together with GA-associated proteins such as P115 and GRASP65 (Nakamura, 2010) maintains the GA structure. GM130 is involved in a variety of important cellular processes including apoptosis (Nakamura, 2010; Chang et al., 2012; Liu et al., 2017). Recent studies have reported that the loss of GM130 in neurons causes GA disruption (Liu et al., 2017). Marra et al. (2007) further demonstrated that loss of GM130 blocks the tethering and fusion of membranes from the endoplasmic reticulum to the GA, resulting in a shortening of the cisternae and breakdown of the Golgi ribbon. In the present study, after HSV-1 infection, the protein and mRNA levels of GM130 decreased to varying degrees, accompanied by the destruction of the GA structure, suggesting that the downregulation of GM130 may be related to GA fragmentation. Furthermore, knocking down GM130 in mock-infected cells resulted in the swelling of the Golgi stack and destruction of the Golgi ribbon, as well as occludin and claudin 5 downregulation and cell apoptosis. However, overexpressing GM130 reduces Golgi fragmentation, ameliorates apoptosis, and partially restores the protein levels of occludin and claudin 5 in HSV-1-infected cells. These results indicate that the GA fragmentation is mainly due to the downregulation of GM130 caused by HSV-1 infection. GM130-mediated Golgi stress is thus involved in the destruction of the BBB.

Apoptosis is a type of programmed cell death, in which a series of caspases such as cleaved-caspase3 are activated in sequence. Caspases primarily cleave cell substrates that are important for maintaining cell life. In an acute cerebral ischemia model, the BBB was destroyed after 30 min of ischemia, manifested by the destruction of claudin 5 and accompanied by the activation of cleaved-caspase 3 in brain endothelial cells. The destruction of claudin 5 was partially blocked by caspase 3 inhibitors (Zehendner et al., 2011). Similarly, Bojarski et al. (2004) found that cleaved-caspase 3 downregulates occludin by cleaving it. Moreover, the caspase cleavage site in occludin was localized to Asp (320) in the C-terminal cytoplasmic domain. In our experiments, we found that HSV-1-infected cells initiate apoptosis and activate a series of caspases, including cleaved-caspase 3. As apoptosis progressed, occludin and claudin 5 protein levels decreased by varying degrees. Furthermore, inhibition of apoptosis and caspase activity partially restored the protein levels of occludin and claudin 5, revealing the involvement of activated caspases in the destruction of tight junction proteins after HSV-1 infection. In addition, previous studies have found that loss of GM130 results in GA destruction and apoptosis of Purkinje neurons in the mouse (Liu et al., 2017). In the current study, the downregulation of GM130, increased the percentage of apoptotic cells and the protein level of cleaved caspase 3, which suggests that GM130 may be involved in apoptosis triggered by HSV-1 infection. Knockdown of GM130 in mock-infected cells resulted in apoptosis and caspase activation, accompanied by a decrease in occludin and claudin 5. We further found that when apoptosis and caspase activation in HSV-1-infected cells was partially inhibited by overexpressing GM130, the downregulation of occludin and claudin 5 were also restored to a certain extent. Interestingly, the protein levels of GM130 were partially restored by using Z-VAD-fmk, which can inhibit caspase activation in HSV-1-infected cells. Similarly, in NRK cells, downregulation of the Golgi-associated protein GRASP65 triggered by staurosporine is inhibited by Z-VAD, which protects GRASP65 from cleaved-caspase 3 cleavage (Lane et al., 2002). Therefore, we believe that the protein levels of GM130 were partially restored by inhibiting caspase cleavage. Although Walker et al. (2004) also found that activated caspases downregulate GM130 protein levels in apoptotic cells, we further observed a drastic decrease in GM130 mRNA levels in HSV-1-infected cells, suggesting that the decrease in GM130 protein levels may be partially due to the reduced synthesis of GM130 caused by HSV-1 infection. Altogether, these results indicate that GM130-mediated Golgi stress is involved in apoptosis, which leads to the destruction of the BBB after HSV-1 infection. Meanwhile, GM130 downregulation is partially due to apoptosis and may be partially due to HSV-1 infection affecting its transcription.

In summary, our experiments revealed that HSV-1 infection triggers Golgi stress, which presents as changes in GA morphology and function. The changes of GM130 expression level regulates Golgi stress response that is involved in endothelial cell apoptosis, which in turn disrupts the structure and function of the BBB. Meanwhile, GM130 downregulation is partially due to apoptosis triggered by HSV-1 infection. These findings indicate that when treating patients with HSE, in addition to conventional antiviral therapy, attention should be given to the protection of important organelles such as the GA and prevention of cell apoptosis. However, our conclusion lacks data *in vivo* to further corroborate. In future studies, we will explore the effect of the GA stress on the BBB in the mouse by injecting HSV-1 H129-G4 intracerebral. How GM130 transcription is affected by HSV-1 infection remains to be elucidated. In addition, whether GM130-mediated Golgi stress also plays a role in other stress conditions is worth studying, such as tumors, neurodegenerative diseases. To the best of our knowledge, this is the first study to link the GA with the BBB during HSV-1 infection. Our results broaden our understanding of the mechanism underlying the BBB disruption in HSE and identify potentially novel targets for the protection of the BBB and therapeutic strategies for treating patients with HSE.

## Data Availability Statement

The raw data supporting the conclusions of this article will be made available by the authors, without undue reservation, to any qualified researcher.

## Author Contributions

WL and QH conceived and designed the study. QH, HL, and CH performed the experiments. QH, HL, and RW analyzed the data. WL and ML contributed reagents, materials and analysis tools. QH wrote the first draft. WL and RW reviewed and revised the manuscript. All authors reviewed the manuscript.

## Conflict of Interest

The authors declare that the research was conducted in the absence of any commercial or financial relationships that could be construed as a potential conflict of interest.

## Abbreviations

BBB: blood-brain barrier
GA: Golgi apparatus
HSE: Herpes simplex encephalitis
HSV-1: herpes simplex virus type 1
TEER: transendothelial electrical resistance.
