# Supplementary material for: Herpes Simplex Virus 1-Induced Blood-Brain Barrier Damage Involves Apoptosis Associated With GM130-Mediated Golgi Stress
Source: Front Mol Neurosci. 2020 Jan 24;13:2. doi: 10.3389/fnmol.2020.00002 (PMC6992570; doi:10.3389/fnmol.2020.00002)
Supplement: Supplementary file 1 [file Data_Sheet_1.docx]

Supplementary original figures

Fig.1A GM130

**
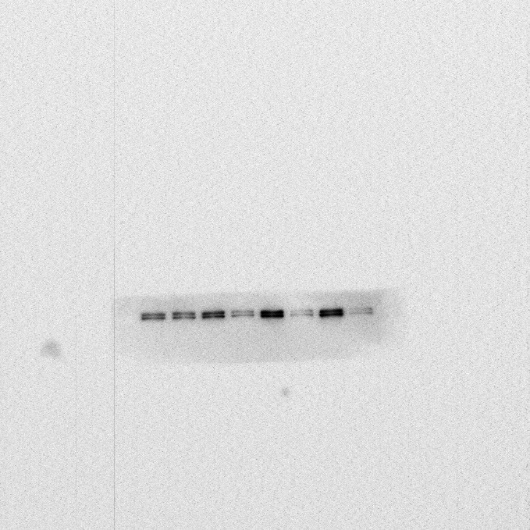
**

Fig.1A cleaved-caspase3

**
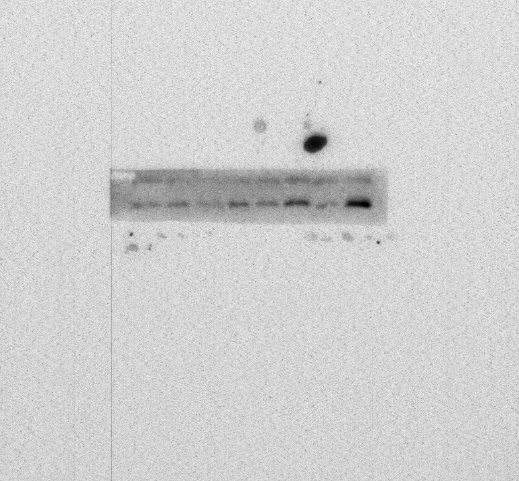
**

**

**

Fig.1A GAPDH



Fig.3A Occludin

Fig.3A Claudin5





Fig.3A GAPDH





Fig.4A GM130


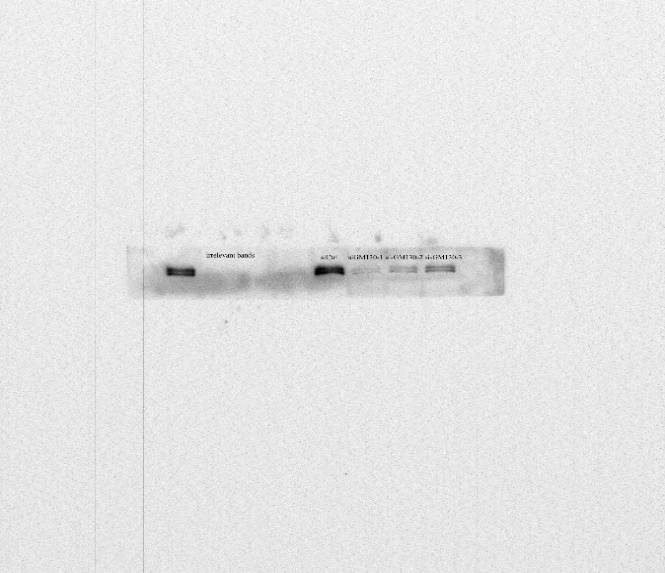


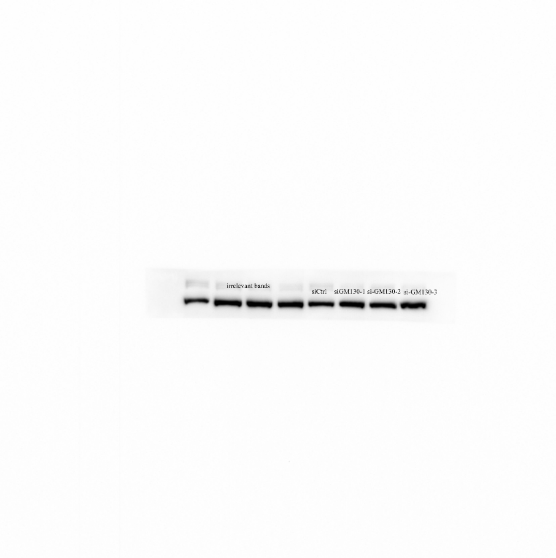


Fig.4A P115


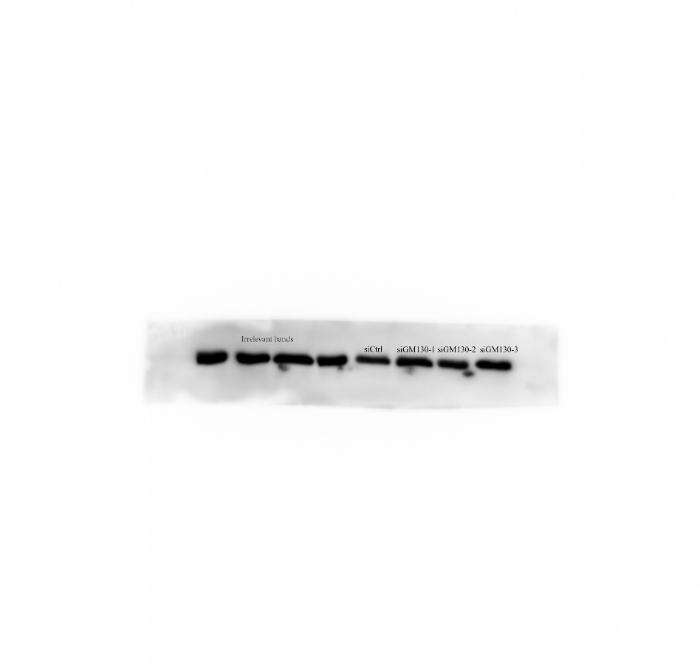


Fig.4A GAPDH

Fig.5B Occludin


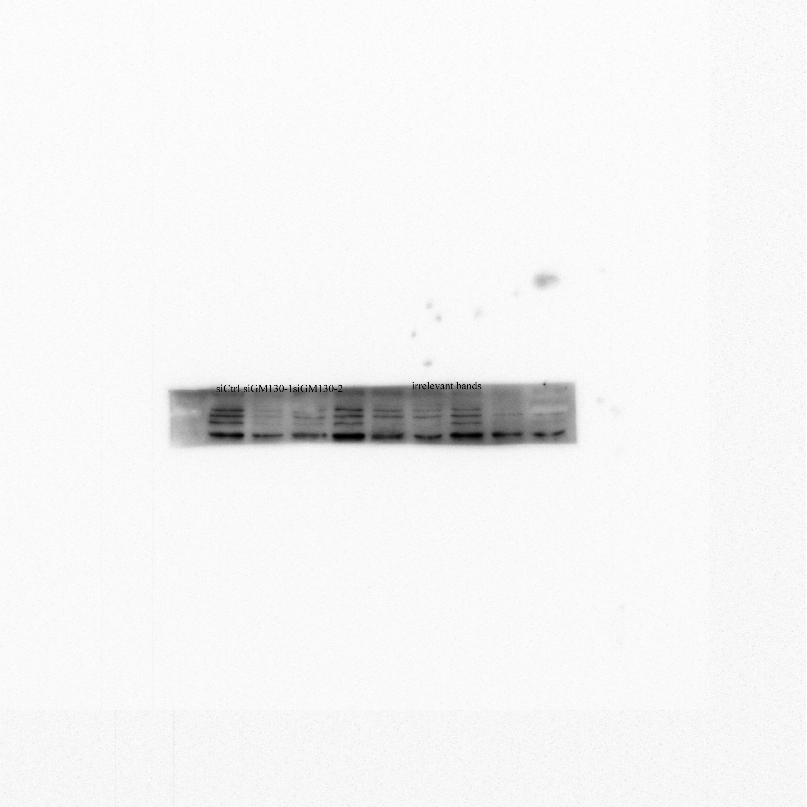


Fig.5B Claudin5


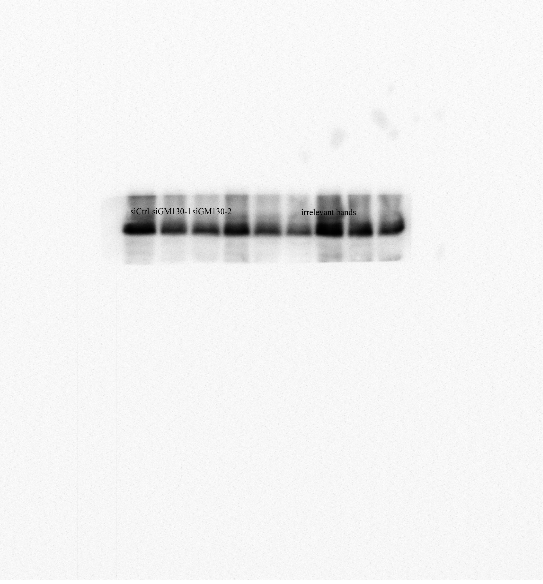


Fig.5B cleaved-caspase3


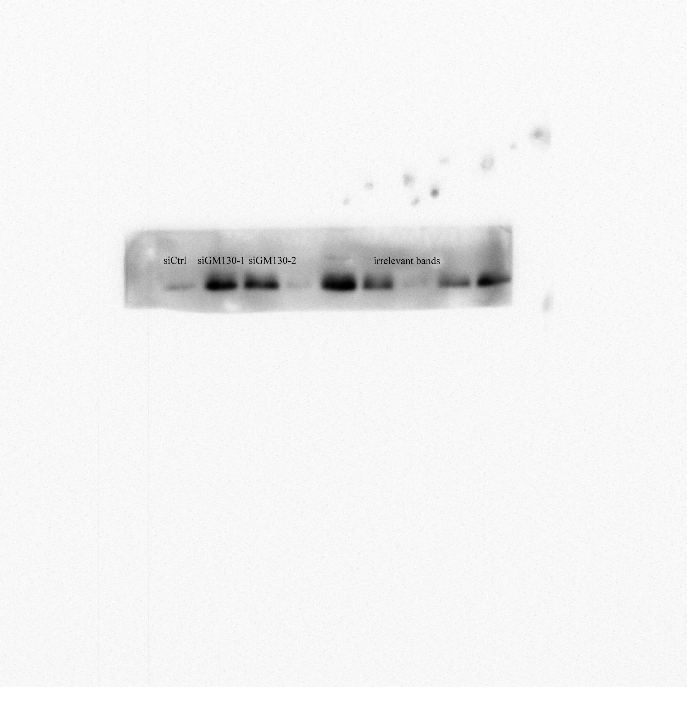


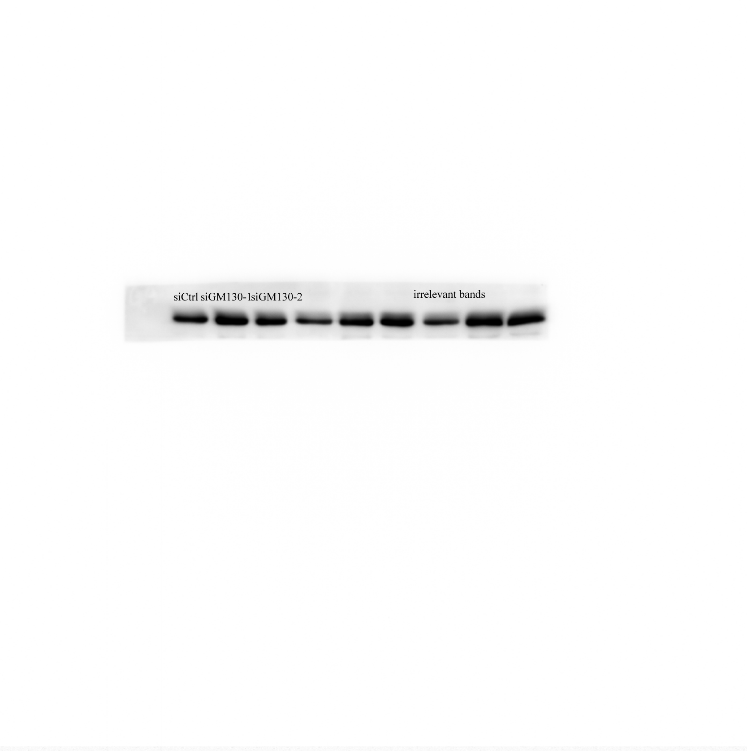


Fig.5B GAPDH

Figure.6A GM130


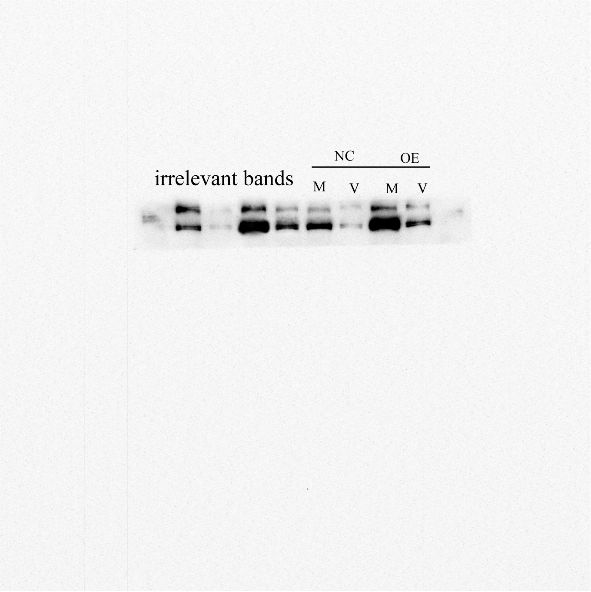


Figure.6A cleaved-caspase3


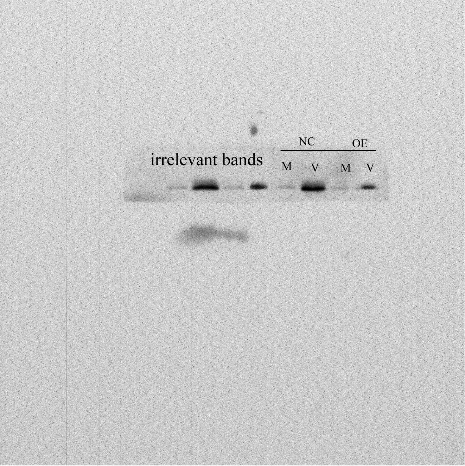


Figure.6A GAPDH


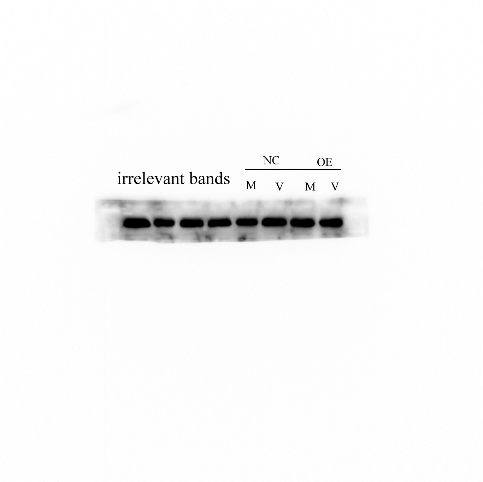


Figure.6D occludin


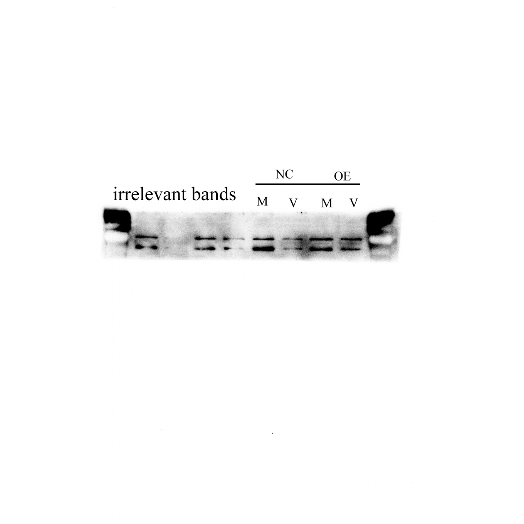


Figure.6D claudin5


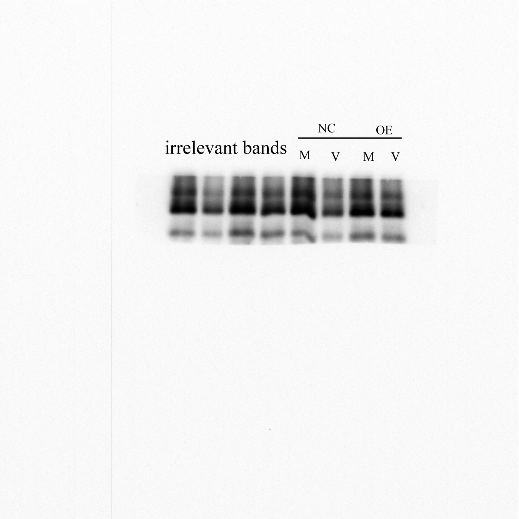


Figure.6D GAPDH


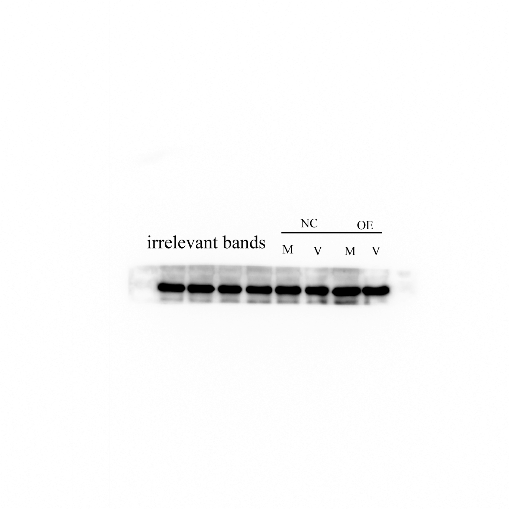


Fig.7A GM130


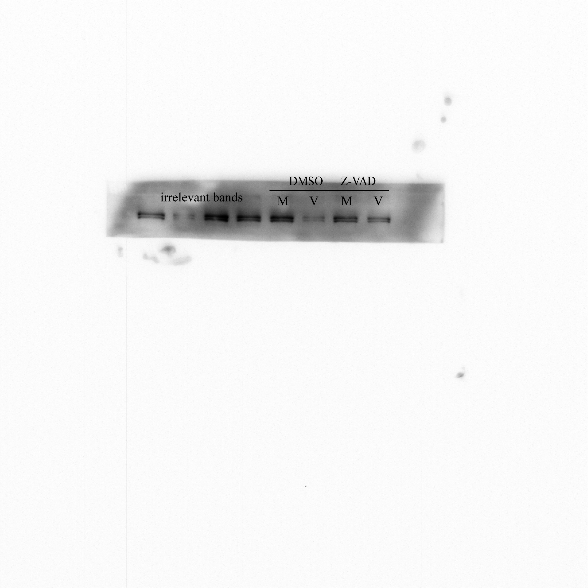


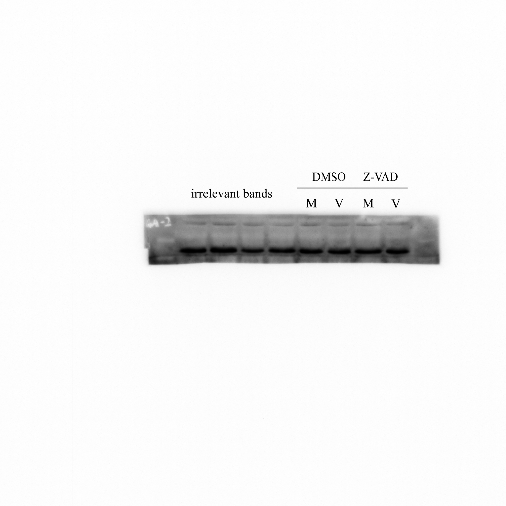
Fig.7A cleaved-caspase3
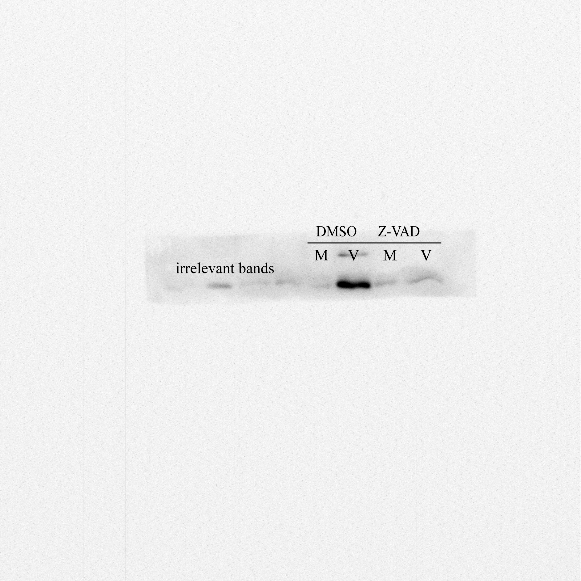


Fig.7A GAPDH

Fig.8A Occludin
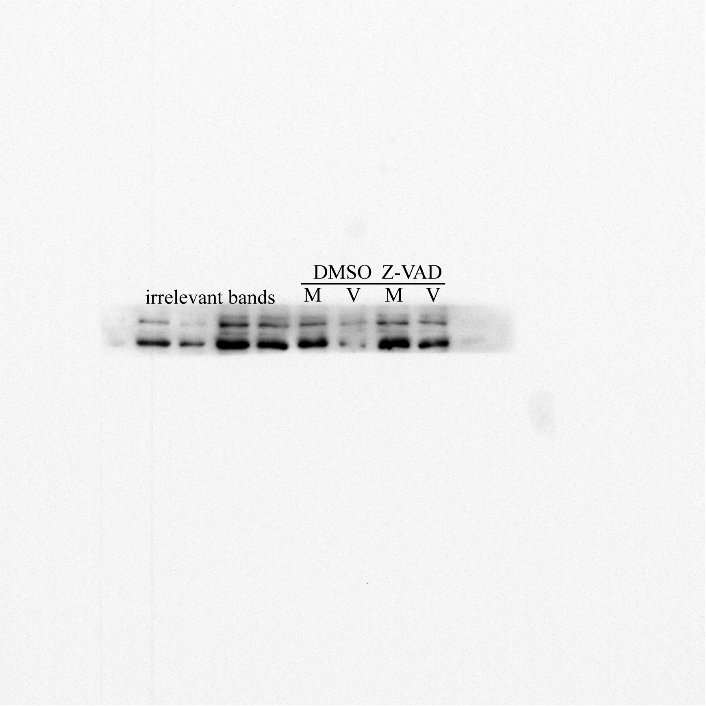


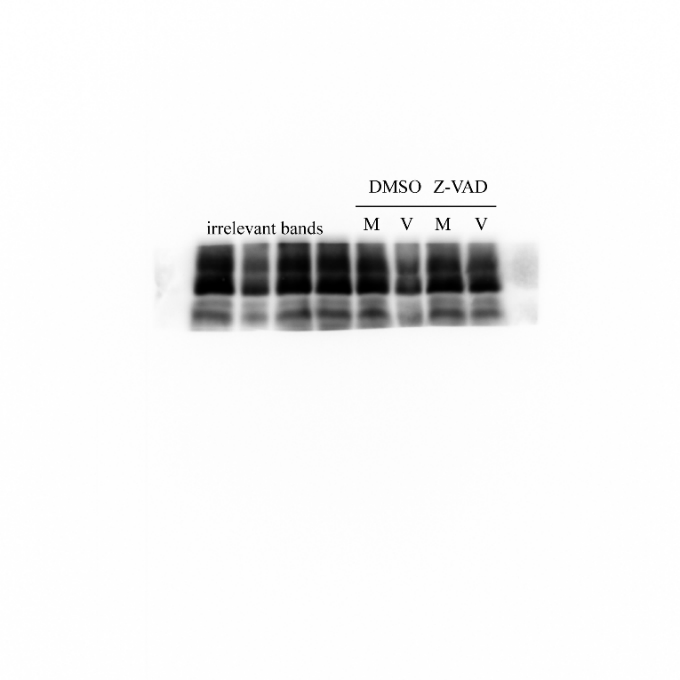


Fig.8A Claudin5
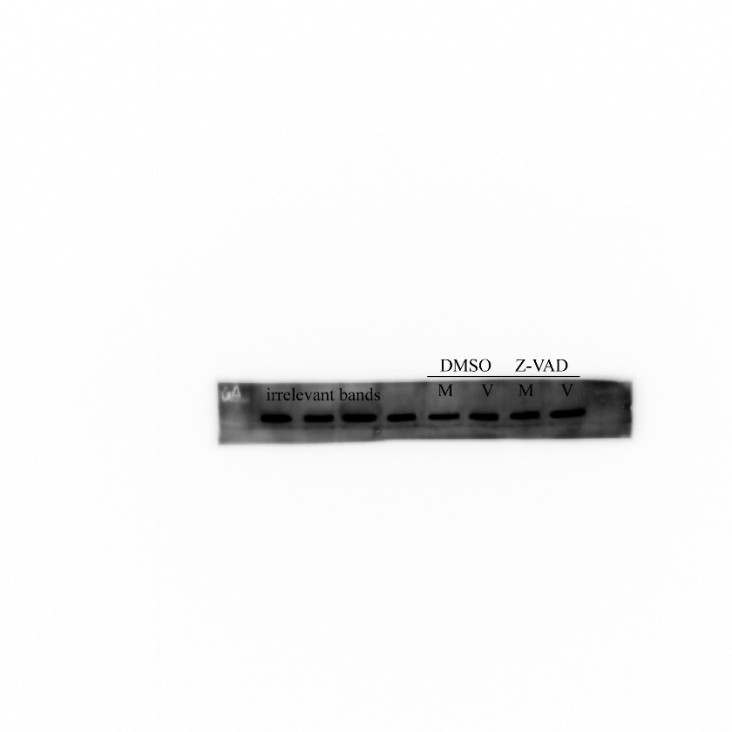


Fig.8A GAPDH
