# Supplementary material for: Herpes Simplex Virus 1-Induced Blood-Brain Barrier Damage Involves Apoptosis Associated With GM130-Mediated Golgi Stress
Source: Front Mol Neurosci. 2020 Jan 24;13:2. doi: 10.3389/fnmol.2020.00002 (PMC6992570; doi:10.3389/fnmol.2020.00002)
Supplement: Supplementary file 2 [file Data_Sheet_2.docx]

Supplementary Material





**Supplementary Figure 1:** Cell viability after siRNA transfection in mock-infected cells**.** Cell viability relative to those in siCtrl -transfected cells was detected by the MTT assay. The data are presented as mean ± SD from three independent experiments and were analyzed by the Student’s t-test.
